# Cardiac Surgery-Associated Acute Kidney Injury: A Continuum from Pathophysiological Mechanisms to Prevention, Management, and Long-Term Care: Short title: Cardiac Surgery-Associated Acute Kidney Injury

**DOI:** 10.31661/gmj.v15i.4284

**Published:** 2026-07-23

**Authors:** Sohrab Negargar, Elnaz Javanshir, Sahar Sadeghi

**Affiliations:** ^1^ Cardiovascular Research Center, Tabriz University of Medical Sciences, Tabriz, Iran

**Keywords:** Cardiac Surgery, Acute Kidney Injury, Cardiopulmonary Bypass, Renal Biomarkers, Perioperative Renal Protection, Renal Replacement Therapy, Long-Term Outcomes

## Abstract

Cardiac surgery-associated acute kidney injury (CSA-AKI) remains a frequent and clinically important complication after coronary artery bypass grafting, valve surgery, and complex aortic procedures. Its development reflects the interaction of pre-existing renal vulnerability with perioperative insults, including renal hypoperfusion, ischemia-reperfusion injury, systemic inflammation, oxidative stress, hemolysis, cardiopulmonary bypass-related injury, and nephrotoxic exposure. Conventional diagnostic markers, particularly serum creatinine and urine output, often identify AKI only after functional decline has occurred, limiting opportunities for early intervention. Novel biomarkers such as NGAL, KIM-1, cystatin C, and TIMP-2/IGFBP7 may improve early risk stratification, although their routine clinical implementation remains limited by cost, availability, and uncertainty regarding biomarker-guided treatment pathways. Current prevention and management strategies therefore rely primarily on early risk assessment, hemodynamic optimization, avoidance of nephrotoxins, careful fluid management, and timely renal support. Importantly, CSA-AKI is increasingly recognized as a continuum extending beyond hospitalization, with increased risks of chronic kidney disease, cardiovascular events, and long-term mortality. This review synthesizes current evidence on the mechanisms, diagnosis, prevention, management, and follow-up of CSA-AKI, while highlighting areas where stronger clinical evidence is still needed.

## Introduction

Acute kidney injury (AKI) is among the most frequent and clinically consequential complications after cardiac surgery, remaining a major challenge in perioperative, nephrology, anesthesiology, and critical care practice [1, 2]. Cardiac surgery-associated acute kidney injury (CSA-AKI) affects approximately 20-40% of patients undergoing cardiac procedures, although reported incidence varies according to AKI definition, baseline patient risk, procedure complexity, and perioperative care practices [1, 3]. Severe CSA-AKI requiring renal replacement therapy (RRT) occurs in approximately 1-5% of patients and is consistently associated with markedly increased short-term mortality, particularly among patients with multiorgan dysfunction or hemodynamic instability [3, 4]. Despite considerable advancements in surgical techniques, cardiopulmonary bypass (CPB) technology, anesthetic protocols, and postoperative intensive care management, the incidence of CSA-AKI has remained persistently high [5, 6].The clinical importance of CSA-AKI extends beyond its immediate postoperative consequences [1]. Even mild and transient elevations in serum creatinine have been associated with prolonged hospitalization, increased healthcare costs, higher rates of mechanical ventilation, and greater risk of in-hospital mortality [3, 7]. More importantly, growing evidence suggests that CSA-AKI may initiate a long-term trajectory toward chronic kidney disease (CKD), end-stage renal disease (ESRD), recurrent cardiovascular events, and reduced long-term survival [1, 8]. This evolving understanding has shifted the perception of CSA-AKI from an isolated perioperative complication to a condition with lasting systemic consequences [1, 9]. The pathogenesis of CSA-AKI is complex and multifactorial [2, 5]. Unlike other forms of hospital-acquired AKI that may result from a single dominant insult, this condition typically arises from the cumulative effects of multiple perioperative stressors [1]. Preoperative patient vulnerabilities including advanced age, diabetes mellitus, chronic kidney disease, anemia, and reduced cardiac function interact with intraoperative factors such as hemodynamic instability, ischemia-reperfusion injury, inflammatory activation, oxidative stress, hemolysis, and non-pulsatile renal perfusion during CPB [2, 5]. Postoperative complications including low cardiac output syndrome, sepsis, fluid overload, and nephrotoxic medication exposure may further exacerbate renal injury [1, 6]. Early diagnosis remains a persistent challenge because traditional markers such as serum creatinine and urine output are often delayed and may fail to identify subclinical kidney injury during the early stages of renal damage [1, 3]. In response, recent research has focused on novel biomarkers including neutrophil gelatinase-associated lipocalin (NGAL), kidney injury molecule-1 (KIM-1), and the combined biomarkers tissue inhibitor of metalloproteinases-2 (TIMP-2) and insulin-like growth factor-binding protein 7 (IGFBP7) to facilitate earlier detection and intervention [4, 10]. Simultaneously, increasing emphasis has been placed on preventive strategies aimed at optimizing perioperative hemodynamics, minimizing nephrotoxic exposure, and improving postoperative renal support [1, 4]. Given the persistent burden of CSA-AKI and its far-reaching consequences, a comprehensive understanding of its pathophysiology, prevention strategies, and management approaches is essential [6]. By evaluating current evidence and recent advances in diagnosis and treatment, this paper aims to provide a comprehensive overview of contemporary approaches to reducing the burden of AKI in cardiac surgical patients. Unlike prior reviews that address CSA-AKI primarily as either a perioperative complication or a biomarker-detection problem, this review frames CSA-AKI as a continuum that begins with preoperative renal susceptibility, evolves through intraoperative and postoperative injury mechanisms, and extends into long-term cardiorenal outcomes. This framework is intended to support a more integrated approach to prevention, diagnosis, management, and post-discharge surveillance.

## Epidemiology and Clinical Significance of CSA-AKI

**Table:** Table1. Epidemiology of CSA-AKI Across Cardiac Procedures

Type of Cardiac Surgery	Estimated CSA-AKI Incidence	Dialysis Requirement	Relative Risk Level
CABG	15-30%	1-2%	Moderate
Valve surgery	20-35%	2-4%	Moderate–High
Combined CABG + Valve surgery	30-50%	3-5%	High
Aortic surgery	40-60%	5-10%	Very High
Repeat cardiac surgery	30-45%	4-8%	High

**Table:** Table2. Timeline of Renal Injury During Cardiac Surgery

Perioperative Phase	Major Mechanism	Typical Clinical Trigger
Preoperative	Reduced renal reserve	CKD, diabetes, contrast
Intraoperative	Hypoperfusion	Hypotension, bleeding
	CPB-related injury	Long bypass time
Immediate postoperative	Low cardiac output	Cardiac dysfunction
ICU period	Sepsis/nephrotoxins	Infection, medications

**Table:** Table3. Common Perioperative Nephrotoxic Exposures

Nephrotoxin	Common Clinical Setting	Renal Risk
Contrast media	Pre-op coronary angiography	Contrast-associated AKI
NSAIDs	Pain management	Reduced renal perfusion
Vancomycin	Post-op infection	Tubular toxicity
Aminoglycosides	Severe infection	Acute tubular necrosis
ACE inhibitors/ARBs	Preoperative medication use	Hypoperfusion risk
Diuretics	Volume depletion	Pre-renal injury

**Table:** Table4. Practical Clinical Risk Factors for CSA-AKI

Operative Phase	Risk Factor	Clinical Relevance	Modifiable?
	Anemia	Reduced oxygen delivery	Yes
	Recent contrast exposure	Additional nephrotoxicity	Yes
	Chronic kidney disease	Reduced renal reserve	Partially
Preoperative	Diabetes mellitus	Microvascular dysfunction	Partially
	Heart failure/low EF	Poor renal perfusion	Partially
	Advanced age	Reduced nephron reserve	No
	Emergency surgery	Limited optimization time	No
	Hypotension	Renal hypoperfusion	Yes
	Hemodilution	Reduced oxygen delivery	Yes
Intraoperative	Blood transfusion	Inflammation/oxidative stress	Yes
	Prolonged CPB duration	Inflammatory injury	Partially
	Aortic cross-clamp duration	Ischemic injury	Partially
	Low cardiac output syndrome	Persistent renal hypoperfusion	Yes
	Sepsis	Systemic inflammatory injury	Yes
Postoperative	Fluid overload	Venous congestion	Yes
	Nephrotoxic drugs	Tubular toxicity	Yes
	Reoperation for bleeding	Hemodynamic instability	Partially

**Table:** Table5. Popular Clinical Prediction Models for CSA-AKI

Model	Variables Included	Primary Use	Strengths	Limitations
Cleveland Clinic Score	Baseline creatinine, CHF, surgery type	Predict dialysis-requiring AKI	Widely validated	Focuses on severe AKI
Mehta Score	Pre/intraoperative variables	Predict postoperative renal failure	Easy bedside use	Moderate accuracy
AKICS Score	Perioperative variables	Broader AKI prediction	Includes intraoperative data	Limited validation
Biomarker models	NGAL, TIMP-2, IGFBP7	Early AKI detection	Detect subclinical injury	Higher cost
Machine learning models	EHR + physiologic trends	Real-time prediction	Higher predictive accuracy	Limited implementation

AKICS:Acute Kidney Injury following Cardiac Surgery; CHF:congestive heart failure; CSA-AKI:cardiac surgery–associated acute kidney injury; EHR:electronic health record; IGFBP7:insulin-like growth factor-binding protein 7; NGAL:neutrophil gelatinase-associated lipocalin; TIMP-2:tissue inhibitor of metalloproteinases 2.

**Table:** Table6. Emerging Biomarkers and Adjunct Diagnostic Tools in CSA-AKI

Test/Tool	What It Detects	Time of Utility	Clinical Role
NGAL	Tubular injury	2-6 hours	Early AKI detection
KIM-1	Tubular injury	Early	Injury assessment
Cystatin C	Early GFR decline	Earlier than creatinine	Functional monitoring
TIMP-2 × IGFBP7	Cellular stress	Very early	Risk prediction
IL-18	Inflammation	Early	Research/advanced centers
Serum creatinine trend	Functional decline	Delayed	Routine monitoring
Urine output	Oliguria	Immediate	Bedside monitoring
Renal ultrasound	Obstruction	When indicated	Rule out post-renal causes
Echocardiography	Cardiac function	When indicated	Evaluate low cardiac output
Medication review	Nephrotoxins	Continuous	Identify reversible causes
Lactate monitoring	Tissue hypoperfusion	Immediate	Assess systemic perfusion

GFR:glomerular filtration rate; IGFBP7: insulin-like growth factor-binding protein 7; IL-18: interleukin 18; KIM-1: kidney injury molecule 1; NGAL: neutrophil gelatinase-associated lipocalin; TIMP-2: tissue inhibitor of metalloproteinases 2.

**Table:** Table7. Perioperative Renal Protection Strategies for Preventing CSA-AKI

Perioperative Phase	Preventive Strategy	Clinical Goal
	Assess baseline kidney function (serum creatinine, eGFR, CKD history)	Identify high-risk patients
	Review nephrotoxic medications	Reduce avoidable renal toxicity
Preoperative	Delay surgery after recent contrast exposure (if feasible)	Minimize cumulative renal injury
	Optimize anemia	Improve oxygen delivery
	Control diabetes and metabolic abnormalities	Reduce perioperative stress
	Optimize intravascular volume status	Prevent hypoperfusion
	Avoid prolonged hypotension	Maintain renal perfusion
	Maintain adequate cardiac output	Preserve renal blood flow
Intraoperative	Use individualized MAP targets	Prevent ischemic injury
	Apply goal-directed fluid therapy	Avoid hypovolemia/fluid overload
	Monitor lactate/perfusion markers	Detect hypoperfusion early
	Minimize CPB duration	Reduce inflammatory exposure
	Maintain adequate hematocrit	Improve oxygen delivery
CPB-related	Reduce hemolysis	Prevent tubular toxicity
	Maintain adequate oxygen delivery during bypass	Prevent medullary hypoxia
	Use biocompatible circuits/filtration strategies	Reduce inflammatory response
	Monitor urine output	Detect early AKI
	Monitor serum creatinine trends	Identify worsening renal function
Postoperative	Optimize cardiac output	Maintain renal perfusion
	Avoid nephrotoxic medications	Prevent secondary injury
	Prevent fluid overload	Reduce venous congestion
	Early infection control	Prevent septic AKI

AKI:acute kidney injury; CKD:chronic kidney disease; CPB:cardiopulmonary bypass; CSA-AKI, cardiac surgery–associated acute kidney injury; eGFR:estimated glomerular filtration rate; MAP:mean arterial pressure.

**Figure-1:**
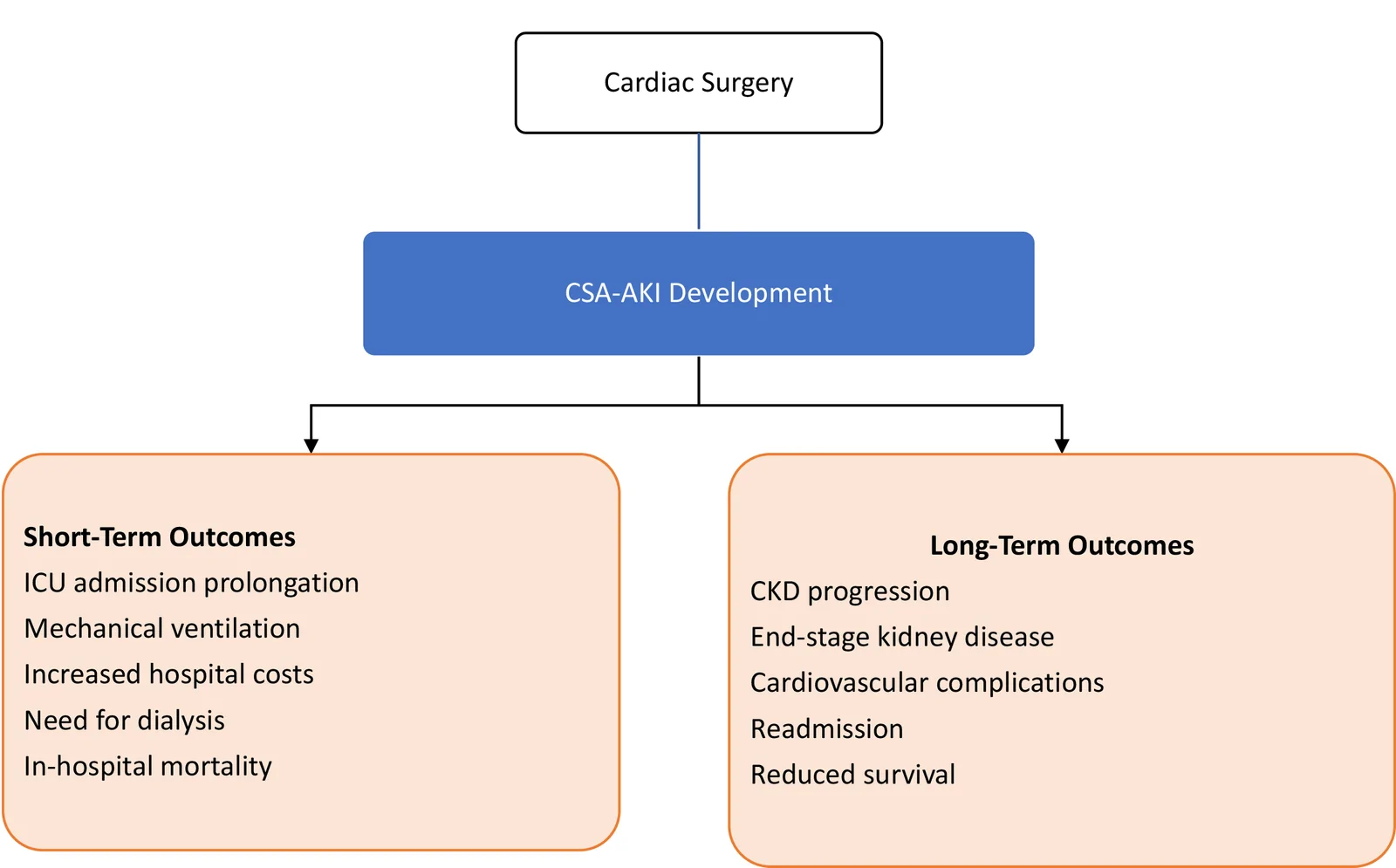
Figure 1. Short-Term and Long-Term Consequences of Cardiac Surgery-Associated AKI

**Figure-2:**
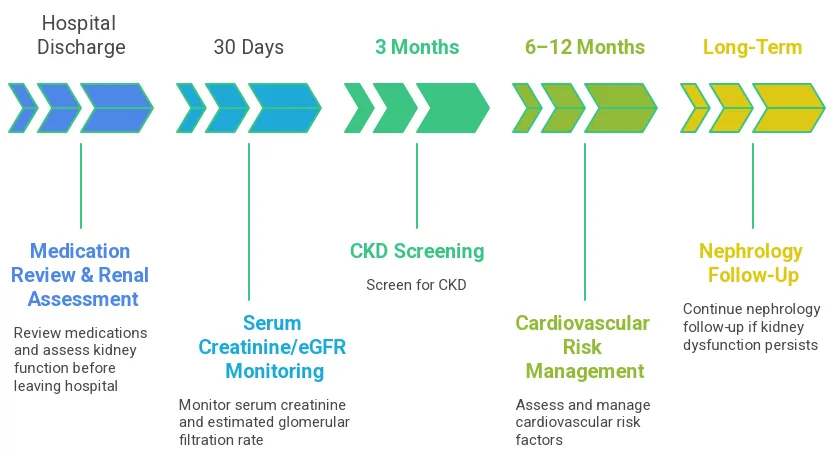
Figure 2. Recommended Follow-Up Timeline After CSA-AKI

CSA-AKI is one of the most common organ-specific complications after cardiac surgery. Its reported incidence varies widely because of differences in patient populations, procedure type, perioperative practices, and AKI definitions [7, 11]. Historically, reported incidence rates differed substantially due to inconsistent definitions of AKI; however, the adoption of standardized classification systems such as the Risk, Injury, Failure, Loss, End-stage kidney disease (RIFLE) criteria, the Acute Kidney Injury Network (AKIN) criteria, and more recently the Kidney Disease: Improving Global Outcomes (KDIGO) guidelines has improved comparability across studies [12, 13]. Current evidence suggests that CSA-AKI occurs in approximately 20–40% of patients undergoing cardiac surgery, making it one of the most frequent organ-specific complications after procedures involving cardiopulmonary bypass (CPB) [1, 7].

Figure-1 summarizes the short-term and long-term consequences of CSA-AKI, including prolonged hospitalization, need for RRT, chronic kidney disease progression, cardiovascular complications, and increased mortality [14, 15]. The incidence may exceed 50% in high-risk populations undergoing complex procedures such as combined valve and coronary artery bypass graft surgeries, aortic arch repair, or repeat cardiac operations [14, 16].

In contrast, minimally invasive procedures and off-pump coronary artery bypass surgery may demonstrate relatively lower rates of renal complications, although findings remain inconsistent [11, 16].

Table1 presents estimated CSA-AKI incidence across common cardiac surgical procedures. Estimates are approximate ranges derived from published observational studies and reviews. Incidence varies according to AKI definition, baseline renal function, surgical complexity, cardiopulmonary bypass exposure, and institutional practice patterns [9, 14, 16]. The severity of CSA-AKI exists along a broad spectrum [1]. Mild forms are often characterized by transient elevations in serum creatinine or brief reductions in urine output, whereas severe AKI may progress to acute renal failure requiring RRT [11, 13]. Approximately 1–5% of cardiac surgery patients require postoperative dialysis, and this subgroup experiences disproportionately poor outcomes, with mortality rates ranging from 40% to 60% [14, 17]. Even among survivors, dialysis-dependent AKI significantly increases the risk of long-term chronic kidney disease (CKD) progression and recurrent hospitalizations [14, 15].

Several demographic trends have contributed to the persistent burden of CSA-AKI [7, 16]. The increasing age of cardiac surgical populations, rising prevalence of diabetes mellitus, hypertension, obesity, and chronic kidney disease, as well as greater utilization of complex surgical interventions, have collectively expanded the population vulnerable to perioperative renal injury [7, 11]. Furthermore, improvements in surgical survival have paradoxically increased recognition of postoperative complications such as AKI among high-risk patients who previously may not have survived surgery [16].

The economic burden of CSA-AKI is equally substantial [9, 18]. Patients who develop AKI often require prolonged intensive care unit (ICU) stays, extended mechanical ventilation, increased use of vasopressors, additional laboratory monitoring, and higher rates of readmission [19]. A large multicenter analysis demonstrated that CSA-AKI significantly increases hospital costs due to longer lengths of stay and greater postoperative resource utilization [9, 18]. These financial implications are particularly relevant in healthcare systems increasingly focused on value-based care and quality improvement metrics [9, 11].

Beyond immediate hospitalization outcomes, CSA-AKI has emerged as a predictor of long-term adverse events [14, 15]. Studies have shown associations between postoperative AKI and increased risks of chronic kidney disease, ESRD, heart failure, recurrent cardiovascular hospitalization, and long-term mortality [14, 15]. Even patients who experience apparent renal recovery may exhibit persistent subclinical nephron loss and heightened susceptibility to future kidney dysfunction [1, 15]. Given its high incidence, substantial economic burden, and long-term clinical consequences, CSA-AKI remains a critical target for perioperative prevention strategies, early diagnosis, and multidisciplinary management [11, 16]. Understanding its epidemiologic trends is essential for identifying vulnerable populations and guiding healthcare interventions aimed at reducing both short- and long-term complications [1, 9].

## Pathophysiology of Acute Kidney Injury in Cardiac Surgery

CSA-AKI is multifactorial and results from a complex interaction between patient-related susceptibility and perioperative renal insults. [1, 20]. Unlike some forms of AKI that may be driven by a single dominant insult, CSA-AKI usually arises from overlapping hemodynamic, inflammatory, ischemic, toxic, and microvascular mechanisms [21, 22]. The kidney is particularly vulnerable during cardiac surgery because of its high metabolic demand, dependence on tightly regulated blood flow, and sensitivity to systemic physiological disturbances [20, 21]. The following mechanisms represent the principal contributors to CSA-AKI development [1, 23]. The temporal relationship between major perioperative phases and dominant renal injury mechanisms is summarized in Table2 [17, 20].

### Hemodynamic Alterations

Renal perfusion is highly dependent on adequate cardiac output, systemic vascular resistance, and mean arterial pressure (MAP) [20, 21]. During cardiac surgery, significant hemodynamic fluctuations frequently occur due to anesthesia-induced vasodilation, blood loss, myocardial dysfunction, arrhythmias, and CPB-related circulatory changes [1, 22]. These events may reduce renal blood flow and compromise glomerular filtration [17, 20]. The renal medulla is particularly susceptible to hypoperfusion because it normally operates under relatively low oxygen tension while maintaining high metabolic activity for sodium reabsorption [21]. Even brief periods of hypotension may precipitate medullary hypoxia and tubular injury [20, 21]. Recent studies suggest that maintaining individualized blood pressure targets rather than uniform MAP thresholds may better preserve renal perfusion in high-risk patients [20, 24]. This suggests that renal protection should not rely solely on fixed blood pressure thresholds but should consider preoperative blood pressure, cardiac function, venous congestion, oxygen delivery, and intraoperative perfusion trends [24]. Low cardiac output syndrome following surgery may further exacerbate renal hypoperfusion by reducing effective arterial circulation, leading to persistent ischemic injury [1, 23]. Additionally, venous congestion resulting from right ventricular dysfunction may elevate renal venous pressure and impair glomerular filtration [17, 20].

### Ischemia-Reperfusion Injury

Ischemia-reperfusion injury (IRI) is a central mechanism in CSA-AKI, particularly during aortic cross-clamping and CPB [1, 20]. During periods of reduced renal perfusion, renal tubular epithelial cells experience ATP depletion, mitochondrial dysfunction, and impaired ion transport [20, 22]. When perfusion is restored, paradoxical injury occurs due to abrupt oxidative stress, calcium overload, endothelial dysfunction, and activation of inflammatory pathways [21, 22]. Reperfusion promotes the generation of reactive oxygen species (ROS), which damage cellular membranes, proteins, and DNA [22]. Mitochondrial dysfunction plays a critical role in IRI progression [20, 22]. Recent experimental research highlights mitochondrial fragmentation and impaired biogenesis as major drivers of persistent renal injury after cardiac surgery [20, 22]. Endothelial injury also contributes to microvascular dysfunction, resulting in sustained tissue hypoxia despite restoration of systemic circulation [21, 22].

### Inflammation and Oxidative Stress

Cardiac surgery induces a systemic inflammatory response that contributes to renal injury. Surgical trauma, blood contact with artificial surfaces during CPB, endotoxemia, and ischemic tissue injury activate innate and adaptive immune pathways [21]. This inflammatory cascade results in the release of: [21, 22] • Tumor necrosis factor-alpha (TNF-α) • Interleukin-6 (IL-6) • Interleukin-8 (IL-8) • Complement activation products Neutrophil-mediated inflammatory mediators

These inflammatory mediators increase vascular permeability, endothelial dysfunction, and tubular apoptosis [21, 22]. Oxidative stress further amplifies kidney injury through excessive production of reactive oxygen species and depletion of endogenous antioxidant defenses [20]. Hemoglobin released during hemolysis may catalyze free radical formation, worsening oxidative tubular damage [25]. Recent studies have explored antioxidant therapies and immunomodulatory interventions, although clinical benefits remain inconsistent [24, 26].

## CPB-Related Mechanisms

Although prolonged CPB duration is consistently associated with CSA-AKI, it may also reflect greater surgical complexity, longer ischemic time, hemodynamic instability, and higher transfusion requirements; therefore, CPB duration should be interpreted as both a potential contributor and a marker of operative risk [1, 22].

CPB introduces several unique mechanisms that increase renal vulnerability [21, 22]. During bypass, blood exposure to non-endothelial artificial surfaces activates complement pathways, coagulation cascades, and inflammatory mediators [21]. Additional CPB-related contributors such as Non-pulsatile renal perfusion, Hemodilution reducing oxygen delivery

, Microembolic phenomena, Hypothermia-related vasoconstriction, and Hemolysis causing free hemoglobin release [1, 22].

Hemodilution during CPB may reduce hematocrit below critical oxygen delivery thresholds, increasing renal medullary hypoxia [21, 22]. Studies have demonstrated an association between prolonged CPB duration and higher CSA-AKI risk [17, 23].

Microemboli composed of platelet aggregates, fat particles, and atheromatous debris may obstruct renal microcirculation, further contributing to ischemic damage [20, 22]. Off-pump coronary artery bypass grafting has been proposed as a strategy to reduce renal injury by avoiding CPB; however, available evidence has shown inconsistent renal benefit, likely because patient selection, surgical expertise, hemodynamic stability, and completeness of revascularization influence outcomes [27].

Nephrotoxic Exposures Perioperative exposure to nephrotoxic agents further contributes to CSA-AKI development [20, 23]. Common perioperative nephrotoxic exposures are summarized in Table3. Their clinical impact depends on baseline kidney function, dose, timing, hemodynamic status, and cumulative exposure to other renal insults [17, 20, 28, 29].

Contrast-associated nephropathy may be particularly important in patients undergoing urgent surgery shortly after coronary angiography [17, 20]. Renin-angiotensin system inhibitors are not directly nephrotoxic, but perioperative continuation may increase the risk of hypotension or reduced glomerular filtration in selected patients [17, 28].

Decisions regarding withholding or restarting these agents should be individualized according to hemodynamic status, renal function, and cardiovascular indication [28].

Postoperative use of vasopressors may also worsen renal vasoconstriction if not carefully titrated [20]. Additionally, inappropriate fluid management may contribute to both hypoperfusion and venous congestion [20, 30].

Recognition of cumulative nephrotoxic exposure has led to growing interest in perioperative “renal stewardship” protocols aimed at minimizing avoidable kidney insults [20, 28].

## Risk Factors and Predictive Models

Clinically, CSA-AKI is best understood as the cumulative result of preoperative vulnerability, intraoperative exposure, and postoperative complications [1, 17]. A structured risk-based approach is therefore essential for identifying high-risk patients, targeting modifiable risk factors, and allocating enhanced monitoring before renal injury becomes clinically apparent [11, 20]. In routine clinical practice, risk assessment begins during the preoperative evaluation [11]. Patients with pre-existing chronic kidney disease (CKD) are among the highest-risk populations due to reduced renal reserve [31]. Baseline serum creatinine, estimated glomerular filtration rate (eGFR), and albuminuria should therefore be assessed before surgery [11, 32].

Advanced age, diabetes mellitus, hypertension, heart failure, peripheral vascular disease, and anemia further increase susceptibility by impairing renal autoregulation and reducing tolerance to ischemic stress [31]. Patients undergoing urgent surgery shortly after coronary angiography may also be exposed to contrast-induced renal injury, which compounds perioperative risk [11, 17].

During surgery, clinicians must monitor factors directly associated with renal hypoperfusion and inflammatory injury [20, 24]. Table4 summarizes common clinical risk factors for CSA-AKI across the preoperative, intraoperative, and postoperative phases [1, 31].

Prolonged CPB duration, extended aortic cross-clamp time, intraoperative hypotension, hemodilution, blood transfusion, and vasopressor dependence are repeatedly associated with higher CSA-AKI incidence [4, 22]. Complex procedures such as combined CABG-valve surgery and aortic surgery carry greater renal risk because of prolonged operative time and greater hemodynamic instability [33, 34]. Postoperatively, renal injury may worsen because of persistent hypotension, low cardiac output syndrome, sepsis, fluid overload, nephrotoxic medications, and prolonged mechanical ventilation [11, 35]. Continuous reassessment in the intensive care unit is therefore essential [11, 24].

Several prediction tools have been developed to estimate the risk of CSA-AKI, particularly severe AKI requiring dialysis [31, 34]. Traditional models such as the Cleveland Clinic Score, Mehta Score, and AKICS Score are clinically useful because they rely on readily available variables [31, 33]. However, many were developed in specific surgical populations and may perform less well when applied to different institutions, contemporary surgical techniques, or broader AKI definitions [33]. Biomarker-enhanced and machine learning models may improve prediction, but most require external validation, cost-effectiveness assessment, and integration into clinical workflows before routine adoption [36, 37]. The strengths and limitations of each model was presented in Table5 [33, 37].

## Diagnostic Challenges and Emerging Biomarkers

Early diagnosis of CSA-AKI remains difficult because conventional criteria primarily detect functional decline rather than early structural kidney injury [38]. In routine practice, clinicians continue to rely on serum creatinine and urine output, as recommended by the KDIGO criteria, yet both markers often detect kidney dysfunction only after substantial renal injury has already occurred [10, 39]. Following cardiac surgery, this limitation becomes even more problematic because hemodilution, fluid resuscitation, diuretic use, and altered muscle metabolism may obscure early renal dysfunction [10, 40]. A clinically important limitation of serum creatinine-based diagnosis is the existence of “subclinical AKI,” in which tubular stress or injury is present before measurable decline in filtration function [41]. This phase may represent a window for intervention, but its clinical management remains uncertain because biomarker positivity does not always translate into progressive or clinically significant AKI [38, 39].

### Conventional Diagnostic Challenges

Serum creatinine remains the most commonly used diagnostic marker because of its widespread availability and low cost [10, 39]. However, it typically rises 24-48 hours after renal injury, making it a delayed indicator [10, 40]. Similarly, urine output can be influenced by diuretics, fluid balance strategies, and hemodynamic interventions, reducing its reliability in postoperative cardiac surgery patients [10, 40]. The KDIGO definition remains the standard clinical framework for AKI diagnosis and is based on changes in serum creatinine and urine output [10, 39]: Increase in serum creatinine by ≥0.3 mg/dL within 48 hours; Increase in serum creatinine to ≥1.5 times baseline within 7 days; or Urine output <0.5 mL/kg/h for at least 6 hours. Despite standardization, these criteria primarily identify functional decline rather than early cellular injury [10, 41].

### Common Causes of Delayed CSA-AKI Diagnosis

Several perioperative factors commonly delay recognition of AKI after cardiac surgery [10, 40]. During cardiopulmonary bypass, hemodilution may temporarily lower serum creatinine concentrations, masking early renal dysfunction [10]. Postoperative aggressive fluid resuscitation may dilute serum biomarkers, while diuretic use may preserve urine output despite ongoing tubular injury [39, 40]. Patients with pre-existing chronic kidney disease present additional diagnostic challenges because even small creatinine elevations may reflect clinically meaningful injury [39, 41]. Furthermore, postoperative mechanical ventilation, vasopressor use, and fluctuating hemodynamics often complicate interpretation of renal perfusion and urine output [10, 40].

## Emerging Biomarkers

Table6 summarizes emerging biomarkers and adjunct diagnostic tools used in the evaluation of CSA-AKI [10, 42]. To overcome the limitations of conventional markers, newer biomarkers and bedside diagnostic tools are increasingly being incorporated into clinical practice [38, 39]. These tools help detect structural kidney injury earlier, identify reversible causes, and guide timely intervention [10, 42]. This integrated approach reflects current clinical practice, where biomarkers complement not replace traditional bedside evaluation [8, 10].

Despite their promise, biomarkers have important limitations [10]. Their diagnostic performance varies across populations, sampling times, assay platforms, and AKI definitions [38, 39]. In addition, biomarker availability and cost may limit routine use, and there is still uncertainty regarding which interventions should be triggered by a positive biomarker result [37]. Therefore, biomarkers should currently be viewed as adjuncts to clinical assessment rather than replacements for hemodynamic evaluation, urine output monitoring, serum creatinine trends, and careful medication review [43].

### Practical Clinical Approach

In contemporary cardiac surgical care, early diagnosis requires combining traditional diagnostic criteria with biomarker-guided surveillance in high-risk patients [10, 43]. Individuals undergoing prolonged cardiopulmonary bypass, complex surgeries, or those with baseline CKD may derive the greatest benefit from enhanced monitoring [38, 42].

## Prevention Strategies

Because CSA-AKI results from multiple interacting insults, prevention requires a bundled perioperative strategy rather than reliance on a single pharmacologic or technical intervention [24, 26]. So, prevention should begin before surgery, continue during intraoperative management, and extend into postoperative intensive care [11]. Current evidence supports a structured strategy centered on maintaining renal perfusion, reducing avoidable nephrotoxic exposures, and identifying high-risk patients early [11, 17]. Table7 summarizes common strategies for preventing CSA-AKI. Preoperative Optimization Prevention begins with careful preoperative risk assessment [11]. Baseline renal function should be evaluated using serum creatinine, eGFR, and prior kidney disease history [11, 17]. Patients with chronic kidney disease, diabetes mellitus, heart failure, anemia, advanced age, and recent contrast exposure require closer monitoring because they have reduced renal reserve [31]. Medication review is particularly important [11]. Medications that may increase perioperative renal risk, including NSAIDs, unnecessary diuretics, and selected renin-angiotensin system inhibitors, should be reviewed before surgery [28]. Decisions should be individualized according to renal function, blood pressure, heart failure status, and urgency of surgery [11, 17]. In elective cases, delaying surgery after recent coronary angiography may reduce cumulative contrast-related injury [17, 20]. Optimization of anemia, glycemic control, and intravascular volume status may further reduce perioperative renal stress [11].

### Intraoperative Prevention

Intraoperative management remains one of the most important opportunities for AKI prevention [24]. Maintaining adequate renal perfusion through avoidance of prolonged hypotension is critical [20, 24]. Patients with chronic hypertension may require higher individualized MAP targets to maintain renal autoregulation and adequate renal perfusion [20]. Goal-directed fluid therapy, cardiac output optimization, and avoidance of prolonged low-flow states are essential[11]. Excessive vasoconstriction should also be avoided because it may worsen renal perfusion [20, 24]. CPB management plays a major role in prevention [22, 24]. Reducing CPB duration, minimizing hemodilution, maintaining adequate oxygen delivery, and limiting hemolysis may reduce renal injury [11, 22]. Several studies have demonstrated higher AKI risk with prolonged bypass time [31, 44]. Multiple pharmacologic interventions have been evaluated for CSA-AKI prevention, including low-dose dopamine, fenoldopam, loop diuretics, statins, sodium bicarbonate, and N-acetylcysteine [24, 26]. However, most have not demonstrated consistent clinical benefit, and routine use solely for renal protection is not recommended [11, 26]. Current evidence supports prioritizing hemodynamic optimization, avoidance of nephrotoxins, maintenance of oxygen delivery, and implementation of structured care bundles [24, 26].

### Postoperative Prevention

Renal protection continues after surgery in the ICU [11]. Early monitoring of urine output, creatinine trends, fluid balance, and hemodynamic status is essential for detecting early kidney dysfunction [10, 43]. Prompt treatment of low cardiac output syndrome, prevention of fluid overload, infection control, and minimizing nephrotoxic medications are critical components of postoperative prevention [20]. In clinical practice, prevention is most effective when viewed as a continuous perioperative bundle rather than isolated interventions [11]. Preoperatively, clinicians should focus on identifying high-risk patients and optimizing modifiable risk factors [11, 17]. Intraoperatively, maintaining renal perfusion and minimizing CPB-related injury are the primary goals [22]. Postoperatively, vigilant monitoring and early correction of hemodynamic instability remain essential to prevent progression from renal stress to established AKI. [10]. KDIGO-based care bundles, including nephrotoxin avoidance, volume optimization, hemodynamic stabilization, glycemic control, and close monitoring of creatinine and urine output, have shown benefit in selected high-risk cardiac surgery populations [11]. However, effectiveness depends on timely implementation, adherence, and appropriate patient selection [43].

## Postoperative Management and Renal Support

Postoperative management plays a decisive role in limiting the progression from early renal stress to established or severe CSA-AKI [10, 11]. Despite appropriate perioperative preventive strategies, many patients develop postoperative renal dysfunction due to persistent hemodynamic instability, fluid imbalance, inflammatory responses, or continued exposure to nephrotoxic agents [20, 24]. In clinical practice, management focuses on early recognition, supportive care, prevention of secondary renal insults, and timely initiation of RRT when necessary [45].

### Early Recognition and Monitoring

The immediate postoperative period is often when AKI becomes clinically evident [10, 11]. Continuous monitoring of urine output remains one of the earliest bedside indicators of worsening renal function and should be routinely assessed in all high-risk patients [43]. Serial serum creatinine measurements are equally important, although clinicians should recognize that creatinine elevation may lag behind actual renal injury [39]. In addition to laboratory monitoring, careful assessment of cumulative fluid balance, serum lactate levels, and invasive hemodynamic parameters helps identify impaired renal perfusion before irreversible damage occurs [24]. Clinicians must also actively search for reversible causes of worsening renal function [17]. Postoperative bleeding, hypovolemia, low cardiac output syndrome, arrhythmias, sepsis, abdominal compartment syndrome, and medication-related nephrotoxicity should all be considered when renal function deteriorates [11, 17]. In centers with access to novel biomarkers, early postoperative testing may help identify subclinical injury before conventional diagnostic criteria are met [43]. When early renal dysfunction is detected, clinicians should rapidly evaluate volume status, cardiac output, venous congestion, bleeding, sepsis, nephrotoxic exposure, and obstruction, because several of these contributors are potentially reversible [17].

### Hemodynamic Optimization

Maintaining adequate renal perfusion remains a cornerstone of postoperative AKI management [20]. Following cardiac surgery, patients may develop myocardial dysfunction, vasoplegia, arrhythmias, or right ventricular failure, all of which may compromise renal blood flow [1, 20]. Appropriate management requires optimization of preload, careful vasopressor titration, and use of inotropic support when indicated [24]. In severe cases of refractory cardiogenic shock, temporary mechanical circulatory support may be necessary to restore systemic perfusion [17]. At the same time, clinicians must avoid excessive vasoconstriction, which may worsen renal ischemia despite improving systemic blood pressure [20, 24]. Individualized hemodynamic targets based on baseline patient characteristics and overall cardiac function are often more effective than rigid blood pressure thresholds [11].

### Fluid Management

Fluid management after cardiac surgery requires careful clinical judgment because both hypovolemia and fluid overload can worsen renal outcomes [20]. Inadequate intravascular volume may exacerbate renal ischemia, whereas excessive fluid administration contributes to interstitial edema, impaired tissue oxygen diffusion, and increased renal venous congestion [43]. Balanced crystalloid solutions are often preferred over chloride-rich fluids in critically ill patients because excessive chloride exposure may contribute to renal vasoconstriction and metabolic acidosis [24]. However, fluid choice should be integrated with the patient’s hemodynamic status, electrolyte profile, and overall resuscitation goals [45, 46].

### Avoidance of Secondary Renal Insults

Once AKI develops, preventing additional kidney injury becomes a major priority [11, 17]. Nephrotoxic medications such as aminoglycosides, nonsteroidal anti-inflammatory drugs, intravenous contrast agents, and unnecessary diuretics should be avoided whenever possible [17]. Early infection control is equally important, as postoperative sepsis remains a major contributor to worsening AKI [20, 35]. Glycemic control, correction of electrolyte abnormalities, and avoidance of prolonged hypotension are also essential supportive strategies that may improve renal recovery [11].

### Renal Replacement Therapy

Patients with severe AKI may require renal replacement therapy when conservative measures fail [45, 46]. Common clinical indications include refractory fluid overload, severe metabolic acidosis, life-threatening hyperkalemia, uremic complications, and persistent oliguria or anuria [46, 47]. Continuous RRT (CRRT) is frequently preferred in hemodynamically unstable cardiac surgery patients because it permits gradual solute clearance and fluid removal with better cardiovascular tolerance than intermittent hemodialysis [45, 48]. The optimal timing of RRT initiation remains controversial [47]. Early initiation may be appropriate in selected patients with progressive fluid overload, worsening metabolic derangements, or persistent oliguria despite optimized hemodynamics. However, routine early initiation in the absence of clear indications has not consistently improved outcomes [45]. Current practice should therefore emphasize individualized decision-making based on clinical trajectory rather than isolated laboratory thresholds [46].

### Multidisciplinary Care and Recovery

Successful postoperative management often requires collaboration among cardiac surgeons, intensivists, nephrologists, pharmacists, and critical care nursing teams [11, 46]. This multidisciplinary approach facilitates early recognition of complications, optimization of treatment strategies, and improved continuity of care [17]. Renal recovery should continue to be monitored even after ICU discharge because some patients experience incomplete renal recovery and remain at increased risk for chronic kidney disease progression [7].

## Long-Term Outcomes and Follow-Up Care

CSA-AKI was historically regarded as a transient postoperative complication, particularly when serum creatinine returned toward baseline before discharge [1]. Contemporary evidence now demonstrates that even apparently reversible CSA-AKI may be associated with long-term renal dysfunction, cardiovascular complications, recurrent hospitalization, and increased mortality [7].This evolving understanding has shifted clinical attention toward post-discharge surveillance and long-term renal care [7, 17].

### Long-Term Renal Outcomes

The severity and duration of AKI strongly influence long-term renal prognosis. Patients with mild and rapidly reversible AKI generally have better outcomes than those with prolonged or dialysis-requiring injury [7]. However, even small postoperative increases in serum creatinine have been associated with accelerated decline in renal function over time [49]. Several cohort studies have demonstrated that patients who develop CSA-AKI are significantly more likely to progress to chronic kidney disease or ESRD, particularly those with pre-existing renal impairment, diabetes mellitus, or repeated episodes of AKI [1, 7]. Persistent structural nephron loss, maladaptive repair mechanisms, renal fibrosis, and microvascular rarefaction may contribute to incomplete recovery and long-term kidney dysfunction [20].

### Cardiovascular Outcomes

The relationship between AKI and cardiovascular disease is increasingly recognized as bidirectional [7, 17]. Patients who develop CSA-AKI frequently experience higher rates of heart failure hospitalization, arrhythmias, myocardial infarction, and cardiovascular mortality after discharge [7]. Persistent inflammation, endothelial dysfunction, fluid overload, hypertension, and neurohormonal activation may contribute to long-term cardiovascular complications [20]. This interaction is often described as part of the cardiorenal syndrome continuum, where kidney dysfunction and cardiac dysfunction progressively worsen one another [7, 17].

### Mortality and Healthcare Utilization

Numerous studies have shown that CSA-AKI is independently associated with increased short-term and long-term mortality [1, 17]. Patients with dialysis-requiring AKI experience particularly poor long-term survival [7, 17]. Even among survivors, recurrent hospitalization rates remain high due to cardiovascular complications, recurrent infections, and progressive renal dysfunction [1, 7]. From a healthcare systems perspective, these long-term complications contribute significantly to readmissions, outpatient dialysis costs, specialist referrals, and reduced quality of life [7, 17].

### Post-Discharge Follow-Up Care

Despite the known risks, many patients receive limited nephrology follow-up after discharge [1, 7]. Structured follow-up programs are increasingly recommended, particularly for patients with moderate-to-severe AKI [7, 17]. Post-discharge care should focus on monitoring renal recovery, identifying progression to CKD, optimizing cardiovascular risk factors, and preventing recurrent kidney injury (Figure-2) [1, 17]. Medication and Lifestyle Management Patients recovering from CSA-AKI often require medication adjustments to avoid recurrent nephrotoxicity [7, 17]. Renin-angiotensin system inhibitors, diuretics, and other medications should be reassessed based on renal recovery [17]. Lifestyle counseling focused on blood pressure control, diabetes management, smoking cessation, weight management, and dietary modification may reduce long-term complications [1, 7].

## Future Directions and Emerging Therapies

Future progress in CSA-AKI will depend on moving from reactive management to precision prevention [1, 7]. This requires improved risk prediction, biomarker-guided interventions, real-time physiologic monitoring, safer CPB strategies, and structured post-AKI recovery programs [7, 20].

### Future Directions in Follow-Up Care

Recent research has emphasized the need for structured survivorship models for AKI patients [1, 7]. Biomarker-guided recovery monitoring, telemedicine follow-up programs, and integrated cardiorenal clinics may improve long-term outcomes [7, 37]. Improved discharge planning and communication between cardiac surgery teams, primary care physicians, nephrologists, and cardiologists remain critical gaps in current care models [1, 17].

### Precision Risk Stratification

Traditional risk prediction models rely primarily on demographic and clinical variables, which may fail to capture dynamic perioperative changes in renal vulnerability [31, 34]. Future approaches are likely to integrate biomarkers, genomics, proteomics, and continuous physiologic monitoring to provide more individualized risk assessment [36, 37]. Machine learning models using electronic health records, intraoperative hemodynamic data, laboratory trends, and biomarker results have demonstrated encouraging predictive accuracy in retrospective and observational studies [34, 36]. However, their clinical value remains uncertain until they are externally validated, shown to improve outcomes, and integrated into workflows without increasing alert fatigue or unnecessary interventions [31, 36].

### Biomarker-Guided Intervention

Although biomarkers such as NGAL, TIMP-2, IGFBP7, and cystatin C have improved early detection of renal stress, their role in guiding therapeutic decisions remains under investigation [10, 39]. Future studies are increasingly evaluating whether biomarker-driven intervention protocols can improve outcomes by triggering early hemodynamic optimization, medication adjustments, and nephrology consultation [37, 43]. The development of multimarker panels may further improve diagnostic precision by distinguishing reversible renal stress from established structural injury [39, 42].

### Novel Pharmacologic Therapies

Multiple pharmacologic strategies are being investigated to directly target mechanisms involved in CSA-AKI [24, 26]. Although mitochondrial protective agents, anti-inflammatory therapies, endothelial stabilizers, antioxidant strategies, and cell-based approaches are biologically plausible, most remain investigational [20, 21, 22]. At present, none has sufficient evidence for routine clinical use in CSA-AKI prevention or treatment [24, 26].

### Advances in Cardiopulmonary Bypass Technology

Technological improvements in CPB systems may reduce renal injury by minimizing inflammatory activation, hemolysis, and hemodilution [21, 22]. Biocompatible circuit materials, improved filtration systems, miniaturized bypass circuits, and enhanced oxygen delivery monitoring may improve renal outcomes during surgery [20, 22]. Future operating rooms may incorporate real-time renal perfusion monitoring systems that allow immediate correction of intraoperative hypoperfusion [7, 24].

### Artificial Intelligence and Real-Time Monitoring

Artificial intelligence (AI) may significantly transform perioperative kidney care by identifying subtle physiologic patterns associated with early renal injury [7, 36]. Continuous integration of hemodynamic monitoring, urine biomarkers, laboratory trends, and electronic health records could allow automated AKI alerts [36, 39]. These systems may eventually support personalized fluid management, vasopressor titration, and individualized renal protection protocols [24]. Post-AKI Recovery Programs Future care models are increasingly recognizing the importance of structured post-discharge surveillance [1, 7]. Dedicated cardiorenal follow-up clinics, telemedicine monitoring, and survivorship programs may help reduce CKD progression and long-term cardiovascular complications following CSA-AKI [7]. Greater collaboration between cardiac surgeons, intensivists, nephrologists, and primary care providers will be essential in these models [17].

### Ongoing Challenges

Despite these promising developments, major barriers remain, including high implementation costs, limited external validation of predictive tools, variability in biomarker availability, and the need for large multicenter trials [36, 37]. Future research should prioritize clinically scalable interventions that can be broadly implemented across diverse healthcare systems [7, 24].

## Conclusion

CSA-AKI is a common and clinically important complication of cardiac surgery that reflects a continuum of preoperative vulnerability, intraoperative renal stress, postoperative injury progression, and long-term cardiorenal consequences. Its multifactorial pathogenesis explains why no single preventive or therapeutic intervention has eliminated risk. Current best practice relies on early risk stratification, optimization of renal perfusion and oxygen delivery, careful fluid and hemodynamic management, avoidance of nephrotoxins, and timely kidney support when severe AKI develops. Emerging biomarkers, artificial intelligence-based prediction models, and advances in cardiopulmonary bypass technology may improve early detection and individualized prevention, but most require further validation before widespread adoption. Importantly, care should not end at hospital discharge, as CSA-AKI survivors remain at increased risk for chronic kidney disease, cardiovascular events, and mortality. A proactive, multidisciplinary, and longitudinal approach is therefore essential to reduce both immediate and long-term complications.

## Conflict of Interest

The authors declare no conflict of interest.

## AI Disclosure Statement

During the preparation of this work, the authors used ChatGPT.com for language editing and grammar improvement. After using this tool/service, the authors reviewed and edited the content as needed and take full responsibility for the content of the publication.
